# Determining the Provenance of Traded Wildlife in the Philippines

**DOI:** 10.3390/ani13132165

**Published:** 2023-06-30

**Authors:** Kate J. Brandis, Phoebe Meagher, Sabine Schoppe, Kyle Zawada, Indira Widmann, Peter Widmann, Roger G. Dolorosa, Roxane Francis

**Affiliations:** 1 Centre for Ecosystem Science, School of Biological, Earth and Environmental Sciences, UNSW Sydney, Sydney 2052, Australia; 2 Taronga Institute of Science and Learning, Taronga Conservation Society, Bradley’s Head Road, Mosman 2088, Australia; 3 Katala Foundation Incorporated, Casoy Road, Purok El Rancho, Santa Monica, Puerto Princesa City 5300, Palawan, Philippines; sabine_schoppe@web.de (S.S.);; 4 Centre for Compassionate Conservation, University of Technology Sydney, Broadway, Sydney 2007, Australia; 5 Puerto Princesa Campus, Western Philippines University, Santa Monica, Puerto Princesa City 5300, Palawan, Philippines; roger.dolororsa@wpu.edu.ph

**Keywords:** stable isotopes, illegal wildlife trade, provenance, pXRF, elemental signatures

## Abstract

**Simple Summary:**

The illegal wildlife trade is a significant threat to biodiversity, with animals being taken from the wild and sold illegally. Often, species with the greatest demand are those that are already threatened, with illegal trade placing even more pressure on these populations. Knowing where traded animals or animal parts have come from is critically important for enforcement, education, and conservation actions. Using three highly traded Philippine species, the Palawan forest turtle, the Philippine cockatoo, and the Philippine pangolin, we developed a novel method of determining the geographic origin of animals or animal parts using a portable X-ray fluorescence device. This method can make significant contributions to combating the illegal wildlife trade through providing a rapid, non-destructive, cost-effective method for identifying the source of traded animals. End users of this tool include enforcement agencies, border force officials, wildlife rescue centres, and conservation researchers.

**Abstract:**

The illegal wildlife trade is a significant threat to global biodiversity, often targeting already threatened species. In combating the trade, it is critical to know the provenance of the traded animal or part to facilitate targeted conservation actions, such as education and enforcement. Here, we present and compare two methods, portable X-ray fluorescence (pXRF) and stable isotope analysis (SIA), to determine both the geographic and source provenance (captive or wild) of traded animals and their parts. Using three critically endangered, frequently illegally traded Philippine species, the Palawan forest turtle (*Siebenrockiella leytensis*), the Philippine cockatoo (*Cacatua haematuropygia*), and the Philippine pangolin (*Manis culionensisis*), we demonstrate that using these methods, we can more accurately assign provenance using pXRF data ($\bar{x}\;$ = 83%) than SIA data ($\bar{x}\;$ = 47%). Our results indicate that these methods provide a valuable forensic tool that can be used in combating the illegal wildlife trade.

## 1. Introduction

The illegal wildlife trade is a significant threat to global biodiversity and a key driver of extinction risk, particularly as many targeted species are already endangered [1,2,3]. The interception of smuggled animals and animal parts is a common occurrence in the enforcement of wildlife trade legislation in many countries [4,5]. The ability to determine where the animal (or part) has come from, its provenance, is essential for conservation organisations, e.g., TRAFFIC, WWF, and IUCN, and enforcement agencies to address the source of the trade, allowing for targeted policy development, enforcement actions [6], repatriation of seized animals [7,8], education, and conservation actions [9,10].

The threat to biodiversity from illegal wildlife trade was formally recognised in 1973 with the signing of the Convention on International Trade in Endangered Species of Wild Fauna and Flora (CITES), an intergovernmental agreement providing the framework for trade in flora and fauna that does not threaten survival of these species. Despite this, the illegal wildlife trade has continued to grow, facilitated by the globalisation of the world’s economy, flexible border arrangements, and improved transportation [11]. Significant declines in elephant *Loxondonta africana* and *L. cyclotis* numbers [12,13] and pangolins (Order: Pholidota) [14] and the extinction of the western black rhino *Diceros bicornis longipes* [15] are all attributed to demand on the illegal wildlife market. Ongoing demand for illegally traded flora and fauna is driven by traditional medicines, exotic pets, and animal parts, e.g., skins, furs [3,16].

In an attempt to alleviate the pressure on wild populations, the captive breeding of some species is permitted as defined in Article 1, paragraph (b) of the CITES convention, [17,18]. Under these circumstances, animals can be legally traded; however, ensuring that animals are not illegally taken from the wild and fraudulently traded as legal, i.e., laundered, is a problem that persists in many regions of the world [19,20,21]. Southeast Asia is recognised as a hotspot for illegally traded wildlife with its high levels of biodiversity, endemic species, and access to unregulated trade routes [11,22]. Our study region, Southern Palawan, Philippines, is a key source of animals in the trade of reptiles, birds, and pangolins [23,24]. Many of these animals are CRITICALLY ENDANGERED with very small populations remaining in the wild [25]. Captive breeding of some of these animals for release aims to bolster wild populations [26,27].

This study focused on three CRITICALLY ENDANGERED endemic Philippine species commonly traded in illegal wildlife markets. The Palawan forest turtle *Siebenrockiella leytensis*, a freshwater turtle endemic to the Province of Palawan [28,29], is the sixth most-confiscated species in the Philippines [30]. Demand for this species as pets and food have resulted in significant numbers being collected from the wild [29,31,32]. Seizure records from 2004–2018 indicated that 23 seizures collected 4723 turtles, with an estimated 1200 also sold illegally in China in 2015 [24]. This species is listed in CITES Appendix II [33], indicating it may become threatened with extinction if trade is not controlled. The Philippine cockatoo *Cacatua haematuropygia* is highly prized as a pet due to its mimicry skills [34]. This species has experienced severe population declines since the 1980s due to habitat loss, persecution, introduced infectious disease, and poaching for the pet trade [35,36]. It is listed in CITES Appendix I (CITES, 2021), indicating it is threatened with extinction and international trade is largely prohibited. The Philippine pangolin *Manis culionensisis* is restricted to the Palawan region, and is one of only eight species of its kind [14]. It is highly sought after for its meat and scales, used in traditional medicines [37]. This species is listed in CITES Appendix I [33]; however, trade is still occurring on a massive scale [37,38].

Using these three globally threatened species at risk from illegal trade, we aimed to test the efficacy of using a portable XRF device (pXRF) compared with stable isotopes (^13^C and ^15^N) as a method for determining provenance, both geographic and source (captive/wild). Portable instruments have the benefit of being more cost-efficient and enable the field collection of data, but they have yet to be applied in the determination of provenance to fight the illegal wildlife trade.

The pXRF collects data on the elemental composition of a sample for ~40 different elements. Provenance determination using element concentration data has been used in a range of fields, including food traceability and safety [39,40], archaeology [41], and conservation [42]. Methods for element measurement can be destructive and expensive, and require laboratories to conduct techniques such as inductively coupled plasma mass spectrometry (ICP-MS). Applications of instruments such as portable X-ray fluorescence (pXRF) devices are providing opportunities to determine animal provenance using rapid, non-destructive, cost-efficient techniques by measuring elemental abundances or concentrations within a sample. X-ray florescence technology includes laboratory-based instruments, e.g., ITRAX [43], or portable instruments [44,45]. Portable instruments have the benefit of being more cost-efficient, require minimal training for data collection, are battery powered, and can be used in a range of locations, e.g., Brazil [46] and Botswana [47], enabling field collection of data.

Elemental signatures, the unique combination of different element concentrations/abundances, are incorporated into tissue through diet, and are a representation of diet during the growth of the tissue. Keratinous tissues, e.g., feathers, scales, and scutes, provide a chemically inert record of diet over time [48] allowing for the study of provenance [48,49,50]. This is critical in determining the source of animals traded in illegal wildlife markets, and can contribute to mapping illegal wildlife trade networks [6,10] and the repatriation of animals [8].

Similarly, stable isotope ratios are determined by diet and can also provide information on provenance, allowing for the identification of geographic source location [51,52] and status as captive or wild animals [53,54]. Stable isotope analysis (SIA) is the measurement of ratios of different isotopes in a sample compared to natural occurrences. Biological studies typically use isotopes of carbon, oxygen, nitrogen, and hydrogen. Previous studies have used isotopes to distinguish between captive- and wild-bred fauna at high accuracies, including mink, frogs, and fish [55,56,57]. Commonly selected stable isotopes for the study of diet and provenance are ^13^C and ^15^N, due to their effectiveness in differentiating populations across a geographical range [53,58] and their direct relation to diet, indicating differences in terrestrial and aquatic diets, and trophic level and niche [59,60,61].

This study aims to demonstrate that pXRF is a valid tool that can be used in determining wildlife provenance, both geographic and source (captive/wild), with the hope that its portable nature will facilitate its use in combating the illegal wildlife trade with uptake by enforcement agencies for use in customs, wildlife markets, and pet stores. It aims to combat the illegal trade by identifying those animals or animal parts that have been fraudulently presented as legally obtained from captive breeding. It will also provide information for targeted enforcement actions [6], for repatriation of seized animals [7], and for education and conservation actions [9,10].

We hypothesise that pXRF data-derived models will perform better than SIA alone due to the quantity of data that can be collected through pXRF. The performance of geographic models compared to source (captive/wild) models will be influenced by the diet, environment, and life history traits of both the captive and wild species of interest.

## 2. Materials and Methods

### 2.1. Study Site and Sample Collection

**Figure 1:**
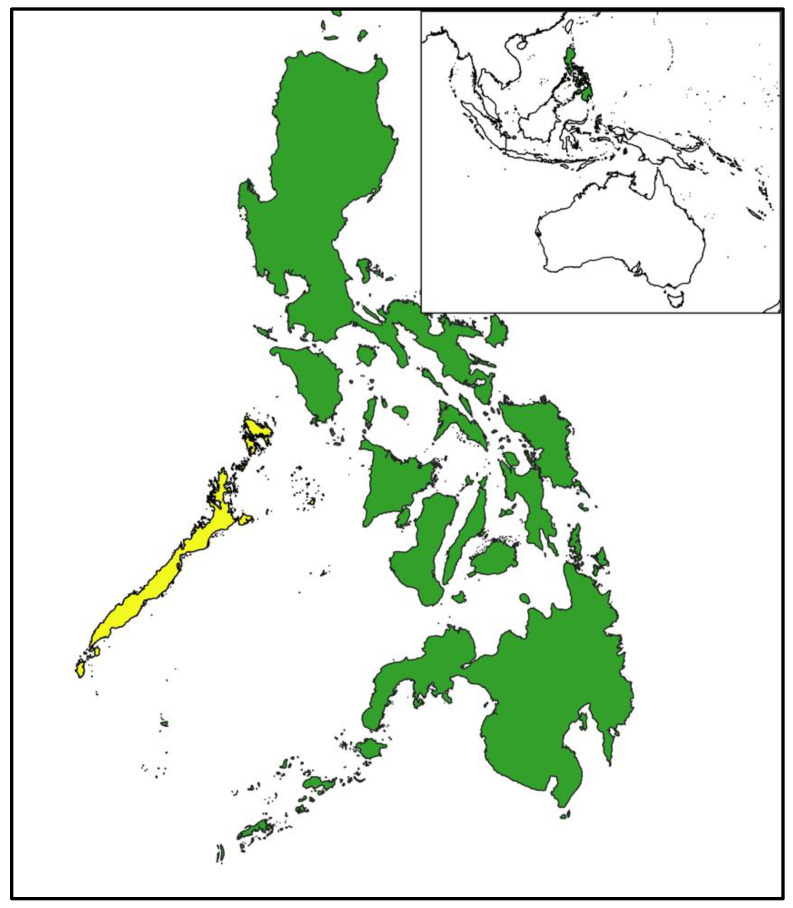
Palawan forest turtles, Philippine cockatoos, and Philippine pangolins were sampled in the Province of Palawan (yellow) in the Philippines (green), situated in Southeast Asia (inset). Due to the ongoing illegal trade of these three species, fine-scale sampling locations are not provided.

Samples from the Palawan forest turtle, the Philippine cockatoo, and the Philippine pangolin were collected by Katala Foundation Incorporated (KFI), Western Philippines University (WPU), and Taronga Conservation Society across Palawan Island, Philippines (Figure 1, Table 1), in 2019. Samples were collected from a total of 45 Palawan forest turtles (24 captive, 21 wild) (Table 1). Captive animals were caught by hand from individual enclosures at the Katala Institute for Ecology and Biodiversity Conservation (KFI) in Narra, Palawan. Wild samples were collected opportunistically during survey field work undertaken by KFI in January 2019. Baited opera traps were set along creeks, semi-submerged, and secured to a nearby tree with rope [62]. Traps were re-checked during the evening and early morning. Scute samples from both captive and wild turtles were collected using a disposable biopsy punch. The edge of the scute was sampled using an upward scraping motion with uniform pressure, producing a long sample of an approximately 3 mm × 20 mm piece of keratin [63]. A single sample was taken from a marginal scute and stored refrigerated in a sealed, labelled cryovial.

Pangolin samples were a mix of scales (n = 6) and nails (n = 12), and all samples came from wild specimens. Scale samples were collected from live animals caught for population surveys. A small ‘v’-shaped notch was cut with a bone cutter and kept individually in a sample vial in a refrigerator. Samples were collected from the lateral, posterior scales, unless there was a damaged scale elsewhere on the animal that could be sampled. One sample per animal was collected. Nail samples were clipped from dead specimens that had been confiscated by the Palawan Council for Sustainable Development Staff (PCSDS) (Philippine government) and kept by the PCSDS in cold storage at their facilities in Irawan, Puerto Princesa City. Scales had been removed from these specimens and were not available for sampling.

Moulted feather samples were collected from within single and multiple bird aviaries at KFI (20 captive samples) and from wild individuals at roost and nest sites of the Philippine cockatoo (45 wild samples) (Table 1). Feathers were also collected opportunistically during routine health and population checks of wild birds. Where possible, feathers were collected from known individuals both captive and wild, i.e., single bird aviaries to avoid re-sampling of the same individuals and banded wild birds. For multi-bird aviaries, potential resampling was limited by only sampling a total of feathers for the total number of birds per aviary, e.g., 3 birds, 3 feathers. While this does not rule out potential resampling of the same individual, we attempted to limit the possibility and the impact it may have on data analyses. Single feathers from each wild roost/nest site were sampled to limit resampling of wild birds.

All samples were classified as being either captive or wild (i.e., animal had been bred or living in captivity for a period of time before sampling, or animal was collected from the wild), and labelled with their geographic provenance (i.e., wild animals collected from distinct regions, and captive animals assigned to their captive facility). Pangolin samples were classified into groups based on geographic location and body part. Although the pangolin sample size was small, we decided to include them in our analyses. Their elusive nature makes the sourcing of larger sample sizes difficult, they are very difficult to breed in captivity [64], and they are at high risk of extinction, making any samples valuable.

This study was conducted under the following permits: CITES permit WT2019–000849, University of New South Wales Animal Care and Ethics Committee number 18/127B, Gratuitous permit 2018–05 from the Palawan Council for Sustainable Development Staff (PCSDS).

### 2.2. X-ray Fluorescence

All samples were scanned for their elemental composition using an Olympus Vanta M-series portable XRF (pXRF, 50 kV, 80 uA) scanner using GeoChem (2) mode, with a 60 s scan time per beam (2 beams, 10 kV and 40 kV). Forty-two elements were measured and all were included in the models.). All samples were scanned within the Olympus Vanta Work Station. Samples were placed over the beam window ensuring the sample completely covered the scan area. This is a non-destructive analysis technique, and the same samples were then used for stable isotope analysis.

### 2.3. ^13^C and ^15^N Stable Isotope Analysis

Feather samples were cleaned in a 2:1 methanol:chloroform wash to remove any surface oils [65], then washed in mild detergent and rinsed three times in reverse osmosis (RO) water before being left to air-dry. Scutes, nails, and scales were cleaned of surface dirt and ground (using a ball grinder or mortar and pestle) to provide a homogenous sample. Samples were weighed into tin caps, and stable carbon (^13^C) and nitrogen (^15^N) isotopes were analysed at the Bioanalytical Mass Spectrometry Facility (BMSF) at the Mark Wainwright Analytical Centre (MWAC) University of New South Wales. A Delta V Advantage Isotope Ratio Mass Spectrometer and Flash 2000 Organic Elemental Analyzer fitted with a Conflo 4 were used.

## 3. Statistical Analyses

XRF beam spectra were analysed using the R package xrftools [66]. To build predictive models, we used the tree-based learning algorithm in the R package XGBoost [67]. We built models with two goals: predicting provenance (captive vs. wild) and predicting geographic location. These models were built for each species separately, in addition to separate models for the elemental pXRF and δ^13^C and δ^15^N isotope ratio data. XGBoost is an implementation of a gradient-boosting decision tree algorithm, which uses sequentially added models to correct errors made by existing models until no further improvements can be made [68]. We used default values of 6 for the maximum depth, 0.3 learning rate, a maximum of 10,000 training rounds, and the ‘multi:softprob’ objective. We used an 80% data split to create the training and test datasets, where the test set data were used to approximate ‘real-world’ model performance. Furthermore, the training data were split again to generate a watchlist dataset (20%) that the XGBoost algorithm assessed during training to avoid overfitting. The models produce probabilities for each class (wild vs. captive, geographical location), which we then converted to a prediction by taking the highest assigned probability as the prediction. To determine model accuracy, we compared the actual classification against the predicted class for the test set data. As model performance could vary based on the random split between training, watchlist, and test data, we ran the train–test process 50 times and aggregated the model performance into mean and standard deviation of accuracy. Variable importance was assessed by looking at the variables’ relative influence on the model outputs. As all pangolin samples were wild, we classified pangolins into 2 groups (Site F/claw or Site G/nail). We ran both geographic and captive/wild classification models on the turtle and cockatoo samples. We removed cockatoo samples that were labelled as wild but were housed within KFI for the testing of geographic origin.

To explore the isotopic differences between wild and captive turtles and cockatoos, we used simple linear models (excluding pangolins as we had no captive samples), separately testing for a significant difference (*p* < 0.05) in δ^13^C and δ^15^N based on their captive or wild origin. Again using separate linear models, we also tested for differences in δ^13^C and δ^15^N in sample geographic provenance for all three species, using the package emmeans to identify sites’ leading differences [69]. As pangolin samples were claws from one location and scales from another, we cannot be sure as to whether we are exploring geographic provenance or body part differences.

## 4. Results

### 4.1. X-ray Fluorescence

**Figure 2:**
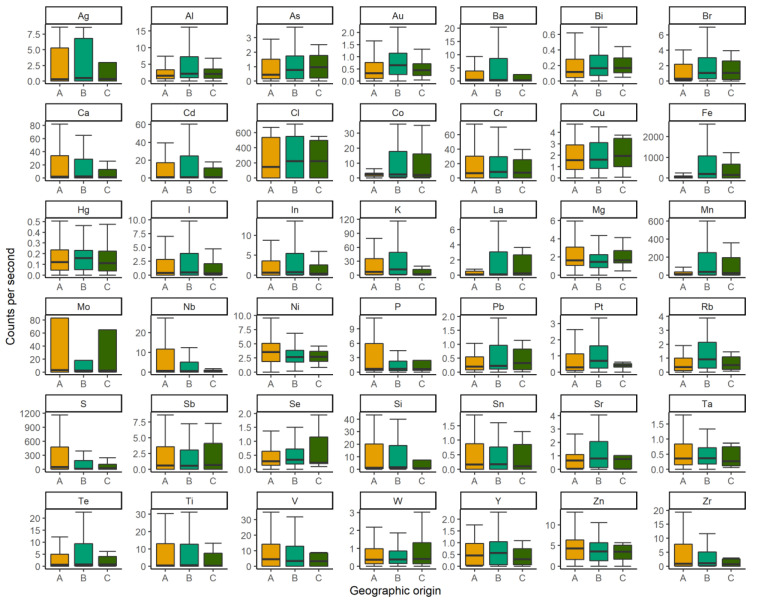
Differences in beam spectra counts per second per element in Palawan forest turtle scute samples collected across the island of Palawan at three locations: KFI (orange, A) and two wild sampling locations (green, B, C).

XGBoost predictive models were successful at differentiating between captive and wild specimens and at predicting geographic provenance using pXRF data (see Figure 2). Models predicted turtle samples as coming from a wild or captive specimen at 88 ± 12% accuracy, largely led by differences in Br, Pb, and Ni (Figure 2) based on scute elemental composition. Geographic provenance (n = 3 locations) was predicted at 94 ± 5% accuracy, led by differences in Br, Zr, and Y.

**Figure 3:**
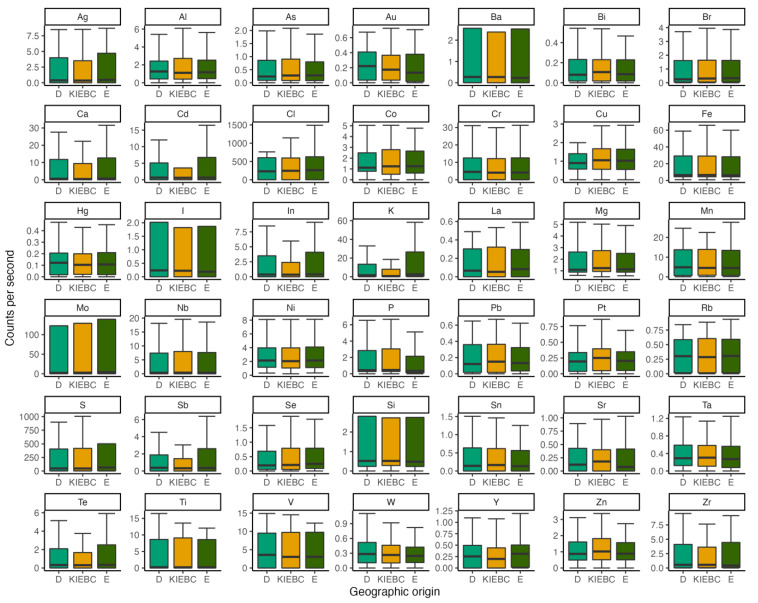
Differences in beam spectra counts per second per element in Philippine cockatoo feather samples collected across the island of Palawan at three locations: KFI (orange, KFI) and two wild sampling locations (green, D,E).

Philippine cockatoo feather classification models predicted geographic provenance (n = 3) at a 62 ± 9% accuracy rate. Key differences were found in the elements In, Mn, and Sr. Classification models also accurately assigned feathers into captive or wild classes with a mean accuracy of 78 ± 2%. Classification was largely led by differences in In, Sr, and Mn, with captive animals having higher concentrations (Figure 3).

**Figure 4:**
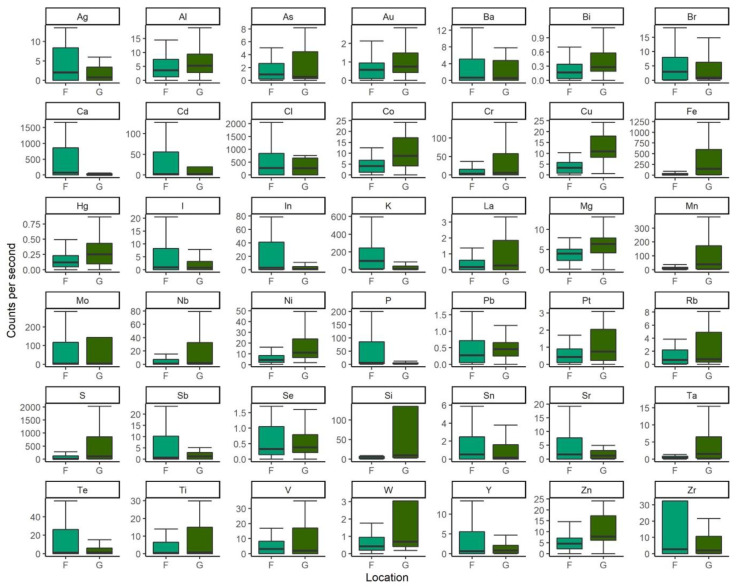
Differences in beam spectra counts per second per element in wild Philippine pangolin scute and nail samples collected across two locations on the island of Palawan (F, G).

Classification models using the elemental composition of pangolin samples were able to accurately predict groups (93 ± 14%). However, due to the samples available, we were unable to determine whether these differences are location- or body part-driven. Classification was largely led by differences in Ca, Zr, and V (Figure 4).

### 4.2. ^13^C and ^15^N Stable Isotopes

**Figure 5:**
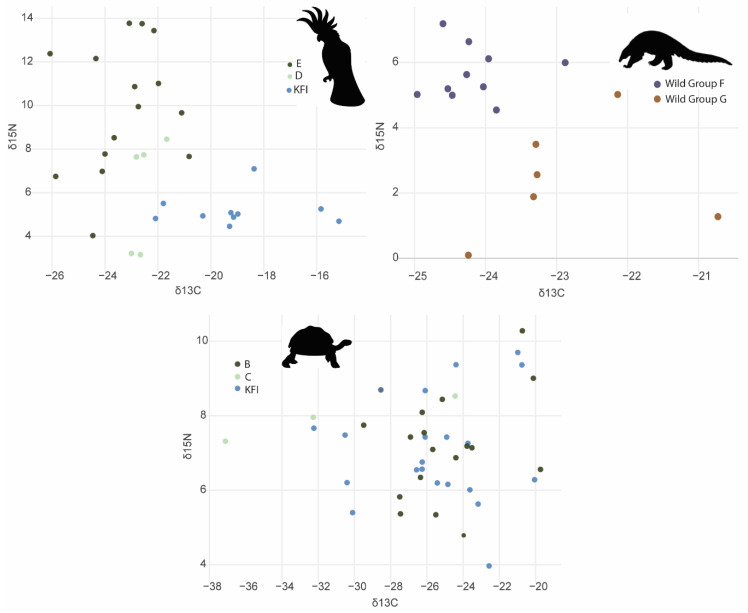
Stable isotope δ^13^C and δ^15^N values for Philippine cockatoo, Philippine pangolin, and Philippine forest turtle with site and wild/captive group status identified.

There were no significant differences in turtle scute δ^15^N (F_(1,42)_ =0.81, *p* = 0.373, R^2^ = 0.02) or δ^13^C (F_(1,43)_ = 0.04, *p* = 0.847, R^2^ = 0.0008) between wild and captive samples. There were no significant differences in turtle scute δ^15^N between sites (F_(2,42)_ = 0.53, *p* = 0.59, R^2^ = 0.02). However, δ^13^C differed significantly between sites (F_(2,42)_ = 3.95, *p* = 0.03, R^2^ = 0.16), led by differences between sites B and C (Figure 5a).

There were significant differences between captive and wild cockatoo feathers in both δ^15^N (F_(1,28)_ = 10.336, *p* = 0.003, R^2^ = 0.26) and δ^13^C (F_(1,28)_ = 12.97, *p* = < 0.001, R^2^ = 0.27) (Figure 5b). Within wild cockatoos, there were also significant differences in δ^15^N (F_(2,22)_ = 4.49, *p* = 0.02, R^2^ = 0.29) when tested for geographic location. There were no significant differences in δ^13^C (F_(2,22)_ = 0.21, *p* = 0.81, R^2^ = 0.02) (Figure 5b).

There were significant differences in Philippine pangolin δ^15^N groups (F_(1,14)_ = 26.7, *p* = 0.0001, R^2^ = 0.65). δ^13^C also differed significantly between groups (F_(1,14)_ = 9.10, *p* = 0.009, R^2^ = 0.35) (Figure 5c).

### 4.3. Predictive Stable Isotope Models

When using XGBoost predictive models and stable isotope results, we achieved 66 ± 16% accuracy predicting the captive or wild source of the Palawan forest turtle, led by δ^15^N, and 73 ± 14% when predicting geographic origin, led by δ^13^C. The models predicted the captive or wild status of the Philippine cockatoo at 28 ± 1% accuracy, led by differences in δ^13^C, and we achieved 27 ± 2% accuracy in predicting geographic provenance, led by differences in δ^15^N. Predictive models using Philippine pangolin stable isotopes performed poorly, with predicted geographic provenance at 41 ± 25% accuracy, led by δ^15^N.

Wild cockatoos from site E had a mean δ^15^N of 9.8‰ compared to site D’s 5.9‰ and captive animals’ 5.22‰ at KFI. Captive cockatoos had a less depleted mean δ^13^C (–19.03‰) than wild cockatoos, with δ^13^C −22.7‰ at site E and −21.93‰ at site D, indicating different sources of carbon.

## 5. Discussion

This study has demonstrated that it is possible to use data derived from a field-portable X-ray fluorescence device to accurately determine both geographic provenance and source (captive/wild) provenance of three Philippine species. Models using beam spectra data derived from pXRF were more accurate than those using stable isotope data (^13^C and ^15^N) for both geographic and source provenance models (Table 2). This suggests that pXRF technology shows promise as a low-cost, rapid, and portable solution for tackling the illegal wildlife trade.

The differences in prediction accuracy observed between the pXRF and SIA techniques are driven by the quantity of data collected through each technique. The ability of the pXRF to measure 42 elements in a single scan provides more sources of unique information compared to two stable isotopes, allowing models to utilise a richer dataset when training. It also uses a field-portable device, which does not require the use of large laboratory equipment, as is required for stable isotope analysis. It has the potential to be used on live animals, provided X-ray safety protocols are followed [70,71], reducing the need to invasively collect samples from animals, e.g., turtle carapaces may be scanned in situ without the need to remove scute samples. It can also be used by enforcement agencies in a range of locations including customs, wildlife markets, and pet stores.

Both the pXRF and SIA techniques measure values in tissues driven by diet [59,60], reflecting the habitat in which they grew the tissue. The species in this study represent a varied group of taxa, birds, mammals, and reptiles, and a range of feeding ecologies, from specialist pangolins [72], to omnivorous turtles [29], to frugivorous cockatoos [73]. These results are supported by other studies in a range of locations, including monotremes in Australia [54], terrestrial and marine waterbird species with diverse diets including piscivores, omnivores, and herbivores in Australia [49], and the southern Indian Ocean [74]. In addition, Buddhachat et al. (2016) successfully used pXRF to distinguish between elephant species, with results driven by differences in geographic location [75]. The study results have demonstrated that our technique is robust to varied feeding ecologies and taxa. The Palawan forest turtle model results (Table 2) were more accurate for the geographic provenance models than the captive/wild models. This was consistent for both pXRF- and SIA-derived data. This may reflect the relatively restricted home range individual turtles inhabit [76], ensuring that diet is representative of the local area only. However, both captive and wild Palawan forest turtles had similar ranges for δ^15^N and δ ^13^C values with large overlap (Figure 5), indicating similar sources of nitrogen and carbon in their diet. This highlights the benefits of using pXRF-derived data, providing a greater range of elements and enabling the detection of differences that otherwise might not be detected using SIA alone.

In contrast to the Palawan forest turtle, the Philippine cockatoo geographic source models performed less accurately than the captive/wild ones (Table 2). These birds have the ability to be wider-ranging than turtles, with the potential to be feeding across a larger geographic range and incorporating diet from a variety of locations and sources. The captive/wild models were more accurate, likely due to larger differences in diets between captive and wild animals, than between different wild locations. KFI cockatoo food sources included chicken feed, string beans, capsicum, and corn (S. Schoppe pers. comm.).

The Philippine pangolin models were limited to ‘Group’, representing both location and body part (claw or scale). While the pXRF predictive models for group performed well (93 ± 14%; Table 2), it is not possible to say whether this is due to locational differences or body part differences. The SIA data on their own did not result in accurate models (41 ± 25%; Table 2). Despite this limitation, the inclusion of these data is important as they represent the first collection of elemental data and add to the limited stable isotope data available for pangolins worldwide [77].

Other limitations of this this study include relatively small sample sizes, due to the limited population sizes of the globally threatened species we studied. Larger samples sizes would likely result in improved model accuracy and lower standard deviations. Another limitation is the potential resampling of individuals when using moulted feathers, particularly from multi-bird aviaries. As discussed in the methods section, we attempted to limit this. However, future studies should be aware of this restriction when using moulted feathers.

While this study achieved relatively accurate models for a range of taxa, the ability to do so for other taxa and species may be influenced by aspects that are diet-driven, e.g., life history traits, dietary breadth, dietary shifts, and home range sizes. Similarly, variations in captive diets and how closely they mimic wild diets will influence the ability to determine source provenance.

The further development of pXRF as a forensic tool, in addition to other tools such as SIA [78], genetics [4], and other molecular approaches [79], has significant potential and could achieve global application through the development of species reference libraries that can be collaboratively contributed to on an ongoing basis, providing data from a range of species and geographic locations.

The ability to predict geographic provenance at high accuracies using pXRF allows for the identification of animals and body parts, e.g., scales, horns, feathers, that are sourced from different geographic regions, e.g., Asian or African species [80]. This is of particular importance when only body parts are traded, which would otherwise require genetic testing, which can be expensive and time-consuming [81]. Knowledge regarding geographic and source provenance also enables anti-poaching and conservation efforts in specific geographic regions [10]. Predicting the captive or wild status of traded animals allows for differentiation between those individuals that were raised in captivity and those that were poached from the wild, facilitating the prosecution of illegal traders.

When combatting such a lucrative global problem as the illegal wildlife trade, it is important to have a range of forensic techniques to accurately identify wildlife as either captive or wild-sourced [54,82]. Furthermore, the ability to identify the location from which wild-sourced animals have been collected allows for the focusing of education [83,84], conservation [10], and enforcement actions [85]. Our study has demonstrated that pXRF provides a method that can achieve both these outcomes, and provides valuable additions to the knowledge of three highly traded Philippine species, contributing to the fight against the illegal wildlife trade and species conservation.

## Figures and Tables

**Table 1:** Sample details of the Palawan forest turtle, Philippine cockatoo, and Philippine pangolin collected across Palawan Island, including captive animals cared for by Katala Foundation Incorporated. True locations are not provided in the interest of protecting these highly traded species.

Common Name	Species Name	Sample	Provenance	Site	n
Palawan forest turtle	*Siebenrockiella leytensis*	Scute	Captive	KFI	24
			Wild	B	18
				C	3
Philippine cockatoo	*Cacatua haematuropygia*	Feather	Captive	KFI	20
			Wild	D	24
				E	21
Philippine pangolin	*Manis culionensis*	Claw	Wild	F	10
		Scale	Wild	G	6

**Table 2:** Predictive model results differed in accuracy amongst the three study species, Palawan forest turtle, Philippine cockatoo, and Philippine pangolin, based on the datasets used (portable X-ray fluorescence (pXRF) or stable isotope analysis (SIA)) and based on the response variable (captive or wild status, geographic origin).

Species	Response Variable	pXRF Model % Accuracy ± SD	SIA Model % Accuracy ± SD
Palawan forest turtle	Captive or wild	88 ± 12%	66 ± 16%
	Geographic origin	94 ± 5%	73 ± 14%
Philippine cockatoo	Captive or wild	78 ± 2%	28 ± 1%
	Geographic origin	62 ± 9%	27 ± 2%
Philippine pangolin	Group	93 ± 14%	41 ± 25%

## Data Availability

The data presented in this study are available on request from the corresponding author. The data are not publicly available due to the study animals being critically endangered.
